# Quantifying Step Count and Oxygen Consumption with Portable Technology during the 2-Min Walk Test in People with Lower Limb Amputation

**DOI:** 10.3390/s21062080

**Published:** 2021-03-16

**Authors:** John D. Smith, Gary Guerra

**Affiliations:** 1 Department of Counseling, Health & Kinesiology, Texas A&M University-San Antonio, 1 University Way, San Antonio, TX 78224, USA; johh.smith@tamusa.edu; 2 Sirindhorn School of Prosthetics and Orthotics, Faculty of Medicine, Siriraj Hospital, Mahidol University, Bangkok 10700, Thailand

**Keywords:** lower-limb prosthesis, wearable activity monitors, metabolic cost, 2MWT, oxygen consumption, actigraphy, accelerometer

## Abstract

Step counts and oxygen consumption have yet to be reported during the 2-min walk test (2MWT) test in persons with lower-limb amputations (LLA). The purpose of this study was to determine step counts and oxygen consumption during the 2MWT in LLA. Thirty-five men and women walked for two minutes as quickly as possible while wearing activity monitors (ActiGraph Link on the wrist (LW) and ankle (LA), Garmin vivofit^®^3 on the wrist (VW) and ankle (VA), and a modus StepWatch on the ankle (SA), and a portable oxygen analyzer. The StepWatch on the ankle (SA) and the vivofit3 on the wrist (VW) had the least error and best accuracy of the activity monitors studied. While there were no significant differences in distance walked, oxygen consumption (VO_2_) or heart rate (HR) between sexes or level of amputation (*p* > 0.05), females took significantly more steps than males (*p* = 0.034), and those with unilateral transfemoral amputations took significantly fewer steps than those with unilateral transtibial amputations (*p* = 0.023). The VW and SA provided the most accurate step counts among the activity monitors and were not significantly different than hand counts. Oxygen consumption for all participants during the 2MWT was 8.9 ± 2.9 mL/kg/min, which is lower than moderate-intensity activity. While some may argue that steady-state activity has not yet been reached in the 2MWT, it may also be possible participants are not walking as fast as they can, thereby misclassifying their performance to a lower standard.

## 1. Introduction

In the United States there are nearly 2 million individuals living with limb loss, and this number is estimated to nearly double by 2050 [1]. For those undergoing amputation as a result of diabetes, there is a five-year mortality rate of nearly 77% [2]. Reports from around the globe have indicated a decline in the rate of major limb amputations [3,4,5,6]. This trend is a result of improved education for patients and clinicians and therapeutic footwear [7]. Still, five-year mortality rates for those undergoing major nontraumatic amputations have shown to range from 52% to 80% [8], and some have reported amputation rates nearly two times higher for males than females irrespective of the cause of amputation [9].

Fortunately, persons with lower-limb loss can often be fit, and they can begin ambulating in a prosthesis within a year [10]. A successful outcome for clinicians and patients alike is viewed as a return to functional independence and restoration of quality of life (QoL). From a prosthesis user’s perspective, mobility in a prosthesis appears to be the single most important contributing factor to QoL [11,12,13,14]. Numerous modern prosthetic technologies, ranging from roll-on liners [15,16,17] and prosthetic sockets [18,19,20,21,22] to powered knee and feet [23,24,25], are available for the prosthetist to provide to a suitable candidate. However, these technologies can incur substantial healthcare costs to the user and third-party payers. Outcome measurements and wearable technologies help evaluate the value of prosthetic treatment, but mainstream adoption still appears to be a challenge [26,27].

Performance-based assessments of walking performance and speed are suitable measures as they relate to overall functional capacity [28]. The 2-min walk test (2MWT) is widely recognized as a reliable and responsive measure for determining prosthesis user walking ability [29]. The standard error of measurement (SEM) and minimum detectable change (MDC), both of which provide important clinically relevant indices, have also been determined for the 2MWT in prosthesis users [30]. In addition, the 2MWT has been chosen as the basis for the determination of wearable accuracy in community-dwelling older persons and those having experience stroke [31,32]. Wearable technology, such as pedometers and accelerometers, can measure walking performance outside the confines of the clinic in real-world patient settings [33,34]. The breadth of activity monitoring scholarship has evaluated the activity of users wearing various prosthetic componentry [35], and the ActiGraph™ GTX3X+, a commercial-grade monitor, has shown promising results at estimating energy expenditure in lower limb prosthesis users depending on where the device is placed [36]. Still, for consumer and commercial grade wearables to be useful, their accuracy must be determined. Underestimation of step counts has been observed by some consumer-grade activity monitors at faster walking speeds, such as the Garmin Vivosmart HR^®^ [37], but this device may still be useful in a population that experiences slower walking speeds.

More recently, the 2MWT has been shown to be predictive of the much longer 6-min walk test (6MWT) [38] and has been encouraged as an easier alternative [30,31,32,33,34,35,36,37,38,39,40,41]. However, even though both tests encourage the patient to cover as much distance as possible, only the 6MWT has evaluated and classified submaximal aerobic capacity [42,43,44]. Previous studies in elderly persons observed oxygen uptake (VO_2_) during the 6MWT that reached moderate-intensity physical activity levels [45,46], and in patients with heart failure, VO_2_ can even approach intense physical activity levels [47]. Using the 2MWT as a proxy of aerobic capacity could be erroneous, as the time needed to reach physiological steady-state or moderate-intensity is typically above three minutes [48]. The 6MWT provides ample time for individuals to reach steady-state physiology. In contrast, the 2MWT may be too short in duration to understand aerobic capacity, but this shorter duration may be better tolerated by lower limb amputees. To the knowledge of the authors, there is a dearth of data exploring VO_2_ during 2MWT in persons with lower-limb amputations (LLA). Measurement of VO_2_ during the 2MWT using portable technology in LLA could help elucidate physiological effort required during this outcome measure.

To date, no studies have quantified step count and oxygen uptake during the 2MWT in LLA. This information could be used to affect rehabilitation objectives and prosthesis provision. The purpose of this study, therefore, was to (1) determine step count and step count accuracy of various activity monitors and (2) oxygen consumption during the 2MWT in LLA.

## 2. Materials and Methods

### 2.1. Participants

This cross-sectional study was approved by the Texas A&M University-San Antonio Institutional Review Board (Log #2017-37) and performed according to the guidelines of the Declaration of Helsinki. Participants were recruited through public peer-support groups in San Antonio, Texas, and surrounding communities between August 2018–February 2020. Data collection was performed after participants consented to the study. A convenient sample of 35 participants (Table 1) signed an informed consent before any data collection. For eligibility criteria in the current investigation, we considered participants with an amputation at the tibial or femoral level on one or both sides. On the contrary, participants were excluded if they relied on any assistive walking devices, such as a wheelchair, crutches, etc. All participants were classified as community ambulators capable of varying cadence according to the Medicare Functional Classification Level (MFCL K3). Participants did not have any uncontrolled cardiovascular or respiratory conditions which would have affected their study performance. Bodyweight was measured using a digital weight scale (Detecto SlimPRO, Webb City, MO, USA) while wearing prostheses and shoes, and height was measured with a stadiometer (Seca 213, Hamburg, Germany).

Stride length was calculated by having meter sticks placed on the ground of the walkway and having participants stand behind a line approximately one meter behind the sticks. An iPad camera using the slow-motion video setting was then turned on, and participants were instructed to walk at a self-selected pace that was not too fast and not too slow, just parallel to the meter sticks. The video was then reviewed, and stride length was measured on the prosthesis side from the first heel contact to the next heel contact on the same leg.

### 2.2. Activity Monitor Placement

**Figure 1:**
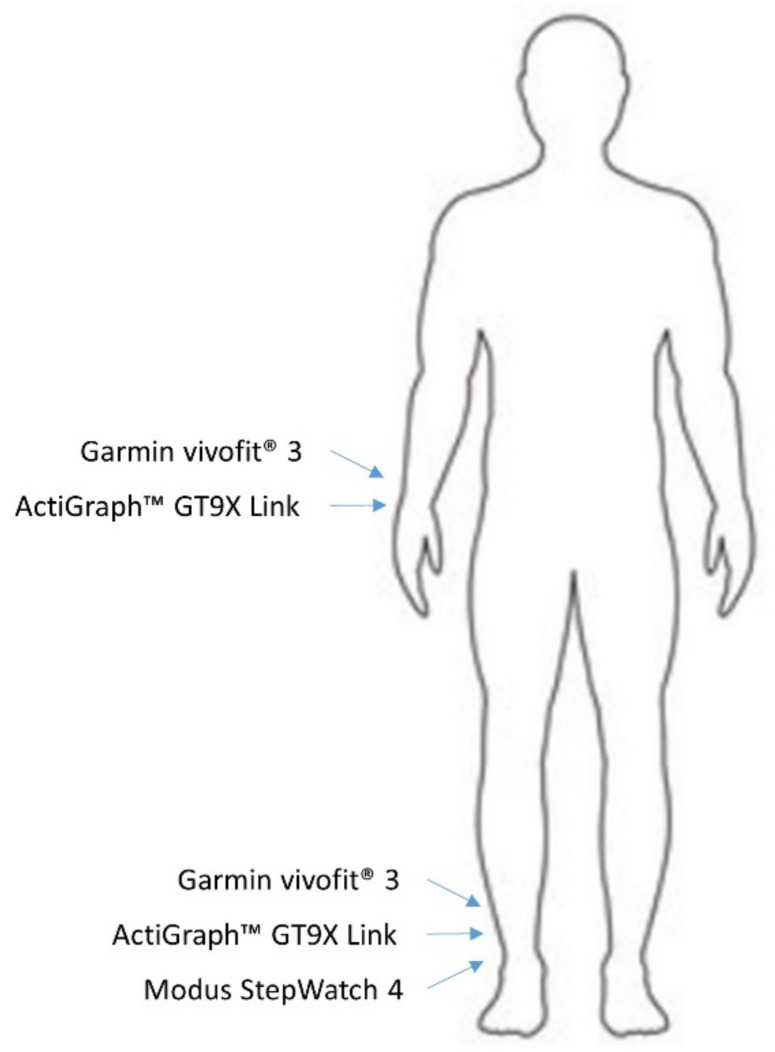
Placement of the activity monitors at the non-dominant wrist and prosthetic ankle.

Participants were then fit with an ActiGraph™ GT9X Link (Pensacola, FL, USA) and Garmin vivofit^®^ 3 (Olathe, Kansas, USA) on the non-dominant wrist with the Link just distal to the vivofit3. A Link and vivofit3 were also fit around the distal prosthesis just above the ankle, with the Link also distal to the vivofit3. Finally, a Modus StepWatch 4 (Edmonds, WA, USA) was also fit inferior to the Link. In the case of those with bilateral amputations, monitors were fit on the non-dominant leg (Figure 1).

While only step counts were of interest, the monitors were still activated using gender, height, weight, and age. The Link monitor was also activated with the location of placement, such as right or left wrist and ankle, with a sample rate of 50 Hz. The Link watch face was also enabled to see step counts. Stride length was entered into the StepWatch application on an iPad, and the “quick” setup was used, which only required height and a yes or no response of two questions: (1) Does the participant frequently run or jog and (2) does the participant show an impaired gait? A 10-stride calibration was then performed to complete the setup.

### 2.3. Metabolic Analyzer

Participants were then fit with a Polar heart rate chest strap and a Cosmed K5 portable metabolic analyzer (Rome, Italy), which was calibrated following the manufacturer’s guidelines. Participants sat for three minutes, during which time the 2-min walk test was explained and demonstrated. Participants were told to walk as fast as possible but to do so safely while covering as much ground as possible. Participants were also told they were allowed to stop but that time would continue and that they were to resume when able to do so. At the end of the walk, they were instructed to stop as quickly as possible and to remain still.

### 2.4. Two-Minute Walk Test

After the three minutes, participants stood behind a start line taped on the floor, and the activity monitors were recorded for pre-step counts, the metabolic analyzer was marked to indicate the start of the walk, and the walk began on a count-down with “three, two, one, go.” Two investigators followed the participant to avoid pacing, one keeping time and filming the lower body and the other with a measuring wheel. Participants walked along a flat, level flooring inside a hallway and made a right turn approximately every 50 m. A one-minute notification was given halfway through the test. At the end of the test, a notification of “five, four, three, two, one, stop” was given. The metabolic analyzer was again marked to indicate the end of the walk, and a rating of perceived exertion (Borg’s 6–20 scale) was recorded. Participants remained still for approximately one minute, after which post-step counts were recorded from the monitors. Distance walked was then recorded, and at a later time, actual step counts (AC) were reviewed and recorded by two investigators using a Grainger standard hand tally counter (Lake Forest, IL, USA). In case AC were not equal between the two investigators, the film was again reviewed by both on separate occasions until AC were determined.

### 2.5. Statistical Analysis

All data were analyzed with IBM SPSS v25 (Chicago, IL, USA). Descriptive statistics were used to report step counts, walking speed, oxygen consumption (VO_2_), heart rate (HR), and rating of perceived exertion (RPE) during the 2-min walk test. Independent *t*-tests were used to make comparisons between sex, and one-way analysis of variance (ANOVA) was used to compare between levels of amputation. For comparisons of the level of amputation, those with right and left transtibial amputations were combined to the unilateral transtibial group, right and left transfemoral amputations were combined to the unilateral transfemoral group and bilateral transtibial for the third independent group. The Bonferroni technique was used to control for type I error during post hoc testing. Alpha was set at 0.05 for all tests.

Repeated-measures analyses of variance (RMANOVA) were used to determine differences between actual step count (AC), Link worn on the wrist (LW), vivofit3 worn on the wrist (VW), Link worn on the ankle (LA), vivofit3 worn on the ankle (VA), and StepWatch worn on the ankle (SA), with Alpha set at 0.05, and partial eta squared reported as the estimate of effect size. The Bonferroni technique was applied for the confidence interval adjustment when examining the pairwise comparisons among the AC and the monitors to maintain significance at 0.05. Cohen’s *d* with the pooled SD was used to compute the effect size between the AC and monitor counts. Single measure intraclass correlation coefficient (ICC) from a two-way random effects ANOVA was used to assess the agreement between AC and monitor counts, with 0.90 or greater considered high agreement; 0.80 to 0.89, moderate agreement; and 0.79 or lower, low agreement [49]. Bland–Altman plots of AC versus monitor counts were used to provide an indication of overrepresentation/underrepresentation of steps (error) and agreement between the monitors [50]. Error scores of zero indicate no difference between the AC registered by the monitors. Scores below zero indicate an overestimation by the monitors, and scores above zero indicate an underestimation by the monitors. These plots illustrate the variability in monitor steps while allowing for the mean difference score and the 95% limits of agreement to be shown. Percent error was calculated as ([steps detected by monitor-AC]/AC) × 100.

## 3. Results

### 3.1. Step Count and Metabolic Variables

Mean step counts, distance walked, VO_2_, HR, and RPE during the 2-min walk test are presented in Table 2. While there were no significant differences in walking speed between sexes, females took approximately 17 more steps than males, *t*_(33)_ = 2.2, *p* = 0.034, resulting in a significantly greater cadence. Oxygen consumption, HR, and RPE were not significantly different between males and females.

There were no significant differences in distance walked, VO_2_, HR, and RPE between amputation levels, *p* > 0.05. Step counts, however, were significantly different between groups (*F*_(2,32)_ = 4.5, *p* = 0.020), and post hoc tests indicated those with unilateral transtibial amputations had significantly greater cadence than those with unilateral transfemoral amputations, *p* = 0.023, Table 3.

### 3.2. Activity Monitors

The RMANOVA indicated significant differences among the step counts during the 2-min walk test, *F*_(5, 29)_ = 4.9, *p* = 0.002, η_p_^2^ = 0.459. Pairwise comparisons indicated LW was significantly lower than AC (*p* = 0.048, *d* = 0.82) and VA was significantly higher than AC (*p* = 0.033, *d* = 0.72). VW (*p* = 1.0, *d* = 0.11), LA (*p* = 0.582, *d* = 0.56), and SA (*p* = 0.996, *d* = 0.09) were not significantly different than AC. Agreement with AC among the monitors during the 2-min walk was highest with SA and VW, while all others indicated very low agreement. Step counts and intraclass correlation coefficients with 95% confidence limits are presented in Table 4.

**Figure 2:**
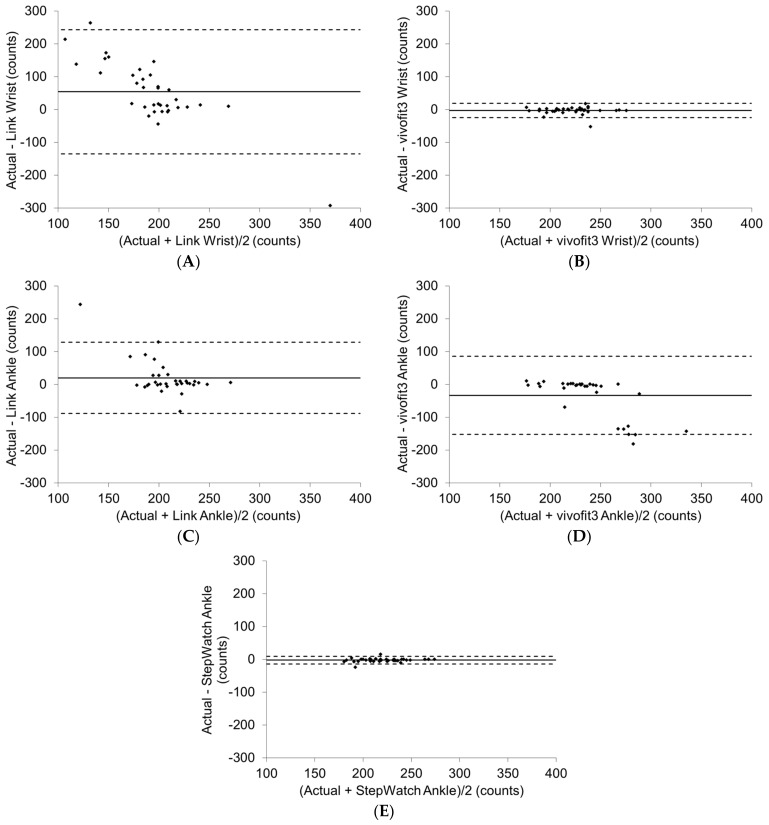
The Garmin vivofit3 worn on the wrist (**B**) and the modus StepWatch worn on the prostheses ankle (**E**) had the least error and tightest limits of agreement compared to the ActiGraph Link worn on the wrist (**A**) and ankle (**C**), and the vivofit3 worn on the ankle (**D**).

Bland–Altman plots are consistent with the ICCs, indicating VW and SA had the tightest limits of agreement (dashed lines representing 2 SD) and least error (closest to zero) compared to LW, LA, and VA (Figure 2).

Percent error was the least in SA, followed by VW, and the greatest in LW (Table 5).

## 4. Discussion

In this study, we measured the step counts in LLA during a 2-min walk test using different activity monitors while oxygen consumption was measured. The SA and VW were the most accurate devices, and oxygen consumption for all participants during the 2MWT was lower than what is considered moderate-intensity activity.

Utilizing performance-based assessments to estimate functional capacity can play a vital role in programs intended to elevate the health and well-being of LLA. Many of these performance-based assessments are field tests, such as the 2MWT, which is designed to utilize minimal equipment, take little time, and is easy to administer. While studies have suggested the 2MWT is a good test of mobility and functional improvement [29,41], little has been done to quantify step counts and oxygen cost, which might be useful information for practitioners or other researchers.

Participants in this study, identified as K3 ambulators, took 220.0 ± 24.3 steps over 147.02 ± 25.9 m during the 2MWT. Although this was farther than the 138 ± 28.5 m walked in 70 recently reported K3 participants [41], the walking speed of our participants aged 48.5 ± 14.8 years is comparable to LLA at ages up to 49 years (77.3 ± 14.8 m) and 50–59 years (74.5 ± 19.5 m) in the same study. While walking speeds of LLA vary greatly across age, sex, amputation level, and duration of amputation, walking speeds in our study were faster [51] and slower [52] than others.

Interestingly, there was no significant difference in the distance walked between sexes. Although females took more steps than males (*p* = 0.034), resulting in significantly greater cadence, this did not impact distance walked between sexes and is most likely due to the shorter stature of females compared to males. This contrasts findings of others who report faster walking speeds for males compared to females [29,41,53]. The resulting similarity in VO_2_ and RPE between men and women suggests the same relative effort during the 2MWT, thus supporting the finding of no difference in distance walked between sexes. This could possibly be a function of the sample such that these male and female participants were similar in mobility and fitness regardless of sex. The current study included only K3 participants, and since studies have explored differences between sexes without categorizing K levels [41,54], comparison across sexes cannot be fully detailed.

When comparing across amputation levels, the unilateral transfemoral group (UTF) took approximately 25 fewer steps than the unilateral transtibial group (UTT). Although the speed and distance walked were not significantly different between groups, the difference in steps can be attributed to the trend of slower speeds, and less distance walked in UTF. Previous studies have also reported slower speeds in this group compared to UTT [41]. Additionally, perceptual efforts were not different between groups, and while VO_2_ was not significantly different, there was a trend for VO_2_ to be slightly higher in UTF, consistent with other findings [55]. While these findings of metabolic and step data shed light on responses during a 2MWT, this information raises further questions when drawing comparisons to previous research. For example, the reason for limb loss [56], type of foot [57], or knee used [58], and prostheses mass [59] are additional variables to consider when drawing conclusions or categorizing performance while using tests such as the 2MWT.

Clinicians and practitioners may be interested in tracking steps in LLA as part of improvement programs. Research-grade activity monitors may not be cost-effective for most. Thus, consumer-grade monitors, such as the Garmin vivofit3, may offer a more affordable alternative. Additionally, there may be a question as to where to place the monitor for a better indicator of activity, such as on the wrist or the prosthesis. To identify the most accurate activity monitor during the 2MWT, this study employed the Link and StepWatch, typically used as research-grade monitors, and the vivofit3 as the consumer-grade monitor.

The most accurate devices in this study were the StepWatch on the ankle (SA) and the vivofit3 on the wrist (VW). These had the greatest agreement with actual steps taken (AC) and the least amount of error, which was below 10% used by some [60] and below 3% recommended by others [61,62]. Our finding of SA accuracy during linear walking is consistent with others [63,64], and the vivofit has been found to be more accurate at faster walking speeds [27] than slower walking speeds [60,65]. It has been shown that error in the vivofit is under 3% at slower walking speeds (53 m/min) and, therefore, a suitable instrument to measure steps during free walking [66]. These authors also found error in the vivofit to be under 1% at speeds of 80 m/min, which is close to the speeds walked and error detected in our study.

Since it is designed to be worn on the wrist, error of the vivofit3 on the ankle (VA) can be explained by its placement and, therefore, not recommended; however, error of the Link on the wrist and ankle (LW and LA, respectively) is more difficult to interpret since previous studies have found it to be accurate [67,68], while others found it to have a greater degree of error at various speeds [66,69]. Differences in findings may be explained by the placement of the device, surface on which participants walk (over ground or treadmill), sampling rate used (for example, 100 Hz as opposed to 50 Hz), population used (healthy, neurological disorder, elderly, etc.), and adjusting the algorithms of the devices.

Participants were instructed to walk as fast and as safely as possible, and this was a self-selected effort. Oxygen consumption during the 2MWT was 8.9 ± 2.9 mL/kg/min, and RPE on Borg’s 6–20 scale was 10.1 ± 2.2 units. This approximates to 2.5 metabolic equivalent of task (METs) and close to a perceived effort as “light”, respectively. Furthermore, HRs during the 2MWT were 102.8 ± 19.4 b/min, and since Borg’s 6–20 scale was designed to reflect HR for each rating point (a rating of 6 equates to 60 b/min, a rating of 20 equates to 200 b/min) [70], this further supports the light effort exerted by the participants. This is below the “moderate” intensity of at least 3 METs and a rating of 12 (close to “somewhat hard”) on Borg’s scale, which are suggested intensities for improvements in fitness [71]. The authors recognize that these metabolic values may not be steady-state, and should participants continue to walk at this pace, VO_2_ may plateau at a slightly higher value. We feel confident, however, that participants may not have been walking to the level of their fitness due to their low perceived effort.

This brings to light that many participants may not be walking faster during the 2MWT due to the limitation of their prostheses or because of the novelty of them performing the test for the first time. Research often employs the 2MWT with a single trial as a means to report functionality. However, these outcomes may be underreported if participants are not truly walking as fast as they can. A previous report found that in a test–retest of the 2MWT, distances walked on the second trial were significantly farther than the first trial [29]. It was suggested this was not due to a learning effect since the practice was given before testing began, nor was it due to a training effect since the duration between tests was negligible. Metabolic and perceptual data in the current study indicate that participants performing the 2MWT may self-select this as a light intensity task and thereby not walk as fast as they can. Due to the lower MET values, it seems that fitness is not a limitation for the 2MWT in K3 participants, and the limitation may simply be a lack of understanding instructions, a perception of their own ability, or choice to walk only as fast as they are comfortable with while wearing the prostheses.

### Limitations

This study is not without its limitations, as we did not explore the role of etiology of amputation on step and physiological variables. As prior scholarship has seen marked differences in walking and physiological measures between dysvascular and traumatic amputees [72], future studies should explore the role amputation etiology plays on the studied variables. Our recruited participants are representative of a convenient sampling of prosthesis users in our community, and future validation studies could perform an a priori sample size estimation to ensure an adequately powered study. Moreover, prior scholarship has observed that successive trials of the 2MWT can increase the distance walked [29]. We did not ask participants to perform multiple 2MWT trials, which may have produced variability in walking distances. The decision not to recruit an able-bodied comparison group, as well as not comparing the 2MWT to 6MWT, are limitations of this current study. The type of prosthesis has been shown to influence step and physiological variables as well. Our analysis did not investigate the role of foot, knee, or prosthetic suspension on outcome measures. Future scholarship should investigate the aforementioned limitations to elucidate further the relationship between amputation cause and prosthesis on step and physiology during the 2MWT.

## 5. Conclusions

To the authors’ knowledge, this is the first study to report step count and metabolic data in LLA K3 ambulators during the 2MWT, which could be useful information used in rehabilitation objectives and prosthesis provision. Researchers, clinicians, practitioners, and others who may use this test should evaluate both walking and physiological measures of their participants and clients to categorize them for mobility and functionality properly. Furthermore, the SA and VW are reliable devices in measuring performance during this test in this sample and, therefore, could be provided with treatment to track changes in cadence and distance walked during the 2MWT across the continuum of prosthetic rehabilitation.

## Figures and Tables

**Table 1:** Participant Characteristics.

Characteristics	Total (N = 35)	Male (n = 19)	Female (n = 16)
Age (years)	48.5 ± 14.8	52.2 ± 14.6	46.5 ± 15.1
Height (cm)	171.0 ± 8.5	176.5 ± 5.7	164.4 ± 6.2
Weight (kg)	86.0 ± 24.6	99.1 ± 21.8	70.5 ± 18.0
BMI (kg/m^2^)	29.2 ± 7.2	31.8 ± 6.9	26.1 ± 6.3
Amputation Duration (y)	9.5 ± 8.7	7.4 ± 5.4	12.1 ± 11.1
Classification			
Right Transtibial	15	11	4
Left Transtibial	8	3	5
Right Transfemoral	2	1	1
Left Transfemoral	7	4	3
Bilateral Transtibial	3	0	3

Data are expressed as mean ± SD. Note: cm; centimeters, kg; kilograms, m^2^; meters square, y; year.

**Table 2:** Two-minute walk test outcome variables by sex.

Parameter	Total (N = 35)	Males (n = 19)	Females (n = 16)
Steps	220.0 ± 24.3	212.1 ± 19.9	229.4 ± 26.2 *
Cadence (steps/m)	110.0 ± 12.1	106.0 ± 9.9	114.7 ± 13.1 *
Distance Walked (m)	147.02 ± 25.9	144.4 ± 24.6	150.2 ± 27.8
Speed (m/min)	73.5 ± 12.9	72.2 ± 12.3	75.1 ± 13.8
VO_2_ (mL/kg/min)	8.9 ± 2.9	8.3 ± 1.6	9.7 ± 3.9
Heart Rate (b/min)	102.8 ± 19.4	98.5 ± 18.7	108.1 ± 19.6
RPE	10.1 ± 2.2	9.7 ± 1.9	10.5 ± 2.4

Data are expressed as mean ± SD. RPE = rating of perceived exertion using Borg’s 6–20 scale, where lower ratings represent lower perceived exertion. * Significantly greater than males. Note: m; meters, mL; milliliters, VO_2_; volume of oxygen, kg; kilograms, b; beats, RPE; rating of perceived exertion.

**Table 3:** Two-minute walk test outcome variables by the level of amputation.

Parameter	Unilateral Transtibial (n = 23)	Unilateral Transfemoral (n = 9)	Bilateral Transtibial (n = 3)
Steps	228.0 ± 21.9	203.2 ± 23.2 *	209 ± 19.9
Cadence (steps/m)	114.0 ± 10.9	101.6 ± 11.6 *	104.5 ± 9.9
Distance Walked (m)	154.1 ± 27.6	136.6 ± 16.0	124.0 ± 10.2
Speed (m/min)	77.0 ± 13.8	68.3 ± 8.0	62.0 ± 5.1
VO_2_ (mL/kg/min)	8.5 ± 3.1	10.6 ± 2.7	8.0 ± 1.2
Heart Rate (b/min)	103.1 ± 18.9	103.7 ± 24.3	96.9 ± 10.4
RPE	10.1 ± 2.2	10.0 ± 2.5	10.0 ± 1.7

Data are expressed as mean ± SD. RPE = rating of perceived exertion using Borg’s 6–20 scale, where lower ratings represent lower perceived exertion. * Significantly greater than Unilateral Transtibial. Note: m; meters, VO_2_; volume of oxygen, mL; milliliters, kg; kilograms, b; beats, RPE: rating of perceived exertion.

**Table 4:** Activity monitor step counts.

Parameter	Step Counts (N = 35)	ICC	95% Confidence Interval
AC	220.0 ± 24.3	-----	----------
LW	165.7 ± 89.4 *	0.005	−0.256 to 0.299
VW	222.8 ± 24.6	0.895	0.802 to 0.945
LA	198.5 ± 47.9	0.111	−0.202 to 0.418
VA	253.2 ± 59.8 *	0.122	−0.141 to 0.398
SA	222.4 ± 23.2	0.967	0.929 to 0.984

Step counts are expressed as mean ± SD. AC is actual counts, LW is Link on the wrist, VW vivofit3 on the wrist, LA is Link on the ankle, VA is vivofit3 on the ankle, SA is StepWatch on the ankle. * Significantly different from AC (*p* < 0.05). ICC = intraclass correlation coefficients.

**Table 5:** Error scores of activity monitors.

Parameter	Percent Error	Mean Error	95% Limit of Agreement
LW	−24.6	54.2 ± 94.5	−134.8 to 243.3
VW	1.3	−2.8 ± 11.0	−24.9 to 19.2
LA	−9.1	20.1 ± 54.4	−88.7 to 128.9
VA	15.1	−33.2 ± 59.5	−152.2 to 85.8
SA	1.1	−2.3 ± 5.7	−13.8 to 9.1

AC is actual counts, LW is Link on the wrist, VW vivofit3 on the wrist, LA is Link on the ankle, VA is vivofit3 on the ankle, SA is StepWatch on the ankle.
